# Growth and Immune Evasion of Lymph Node Metastasis

**DOI:** 10.3389/fonc.2018.00036

**Published:** 2018-02-21

**Authors:** Dennis Jones, Ethel R. Pereira, Timothy P. Padera

**Affiliations:** ^1^Edwin L. Steele Laboratories for Tumor Biology, Department of Radiation Oncology, MGH Cancer Center, Massachusetts General Hospital, Boston, MA, United States; ^2^Harvard Medical School, Boston, MA, United States

**Keywords:** lymphatics, lymph node, tumor, metastasis, immunity

## Abstract

Cancer patients with lymph node (LN) metastases have a worse prognosis than those without nodal disease. However, why LN metastases correlate with reduced patient survival is poorly understood. Recent findings provide insight into mechanisms underlying tumor growth in LNs. Tumor cells and their secreted molecules engage stromal, myeloid, and lymphoid cells within primary tumors and in the lymphatic system, decreasing antitumor immunity and promoting tumor growth. Understanding the mechanisms of cancer survival and growth in LNs is key to designing effective therapy for the eradication of LN metastases. In addition, uncovering the implications of LN metastasis for systemic tumor burden will inform treatment decisions. In this review, we discuss the current knowledge of the seeding, growth, and further dissemination of LN metastases.

## Introduction

**Figure 1:**
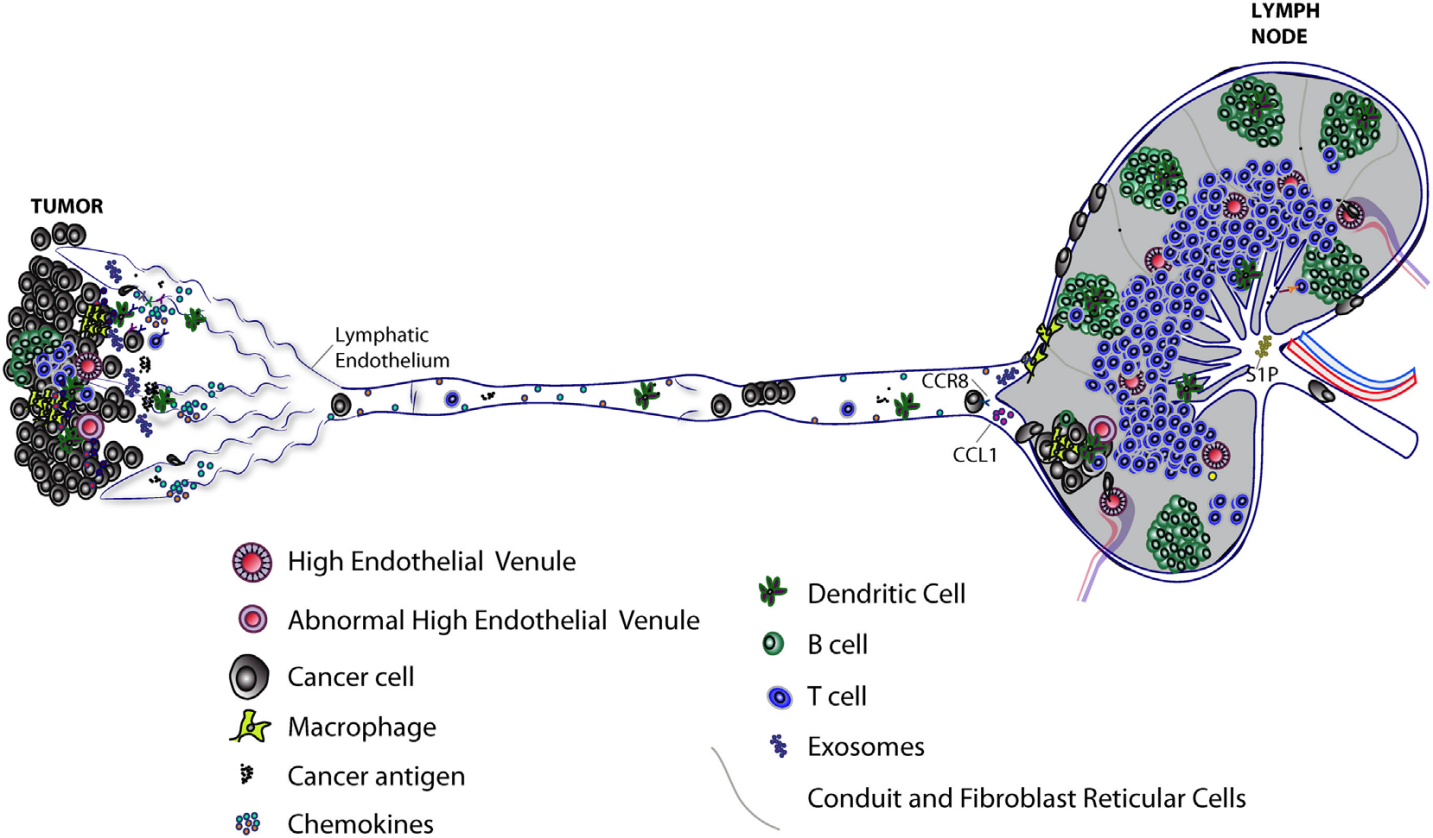
Progression of lymphatic metastasis from primary tumor to tumor-draining LN (TDLN). Primary tumors induce lymphangiogenesis to facilitate lymphatic metastasis and release of immunomodulatory molecules, including exosomes, which lead to immunosuppression of TDLNs. Lymph node (LN) lymphatic endothelial cells (LECs) capture tumor antigen and tolerize T cells *via* programmed death-ligand 1 expression. Tumor-associated lymphatic vessels and tertiary lymphoid organs have been implicated in immune suppression and immune activation. High endothelial venules found in primary tumors can allow infiltration of naive T cells that may further differentiate into effector T cells. Tumor-associated lymphatic vessels recruit both cancer cells and immune cells by releasing chemoattractants (see Figure 2). Cancer cells, T cells, and dendritic cells enter lymphatic capillaries and migrate through collecting lymphatic vessels to LNs. Cancer cells in lymphatic vessels can attach to the lymphatic endothelium en route to LNs. Active mechanisms, such as CCL1/CCR8 signaling, control cancer cell entry into the LN. Polyclonal cancer cells proliferate to form a metastatic lesion that invades deeper into the LN parenchyma, where it can grow and replace LN tissue in the absence of new blood vessel growth. The immune response to a growing metastatic lesion is limited; some immune cells are excluded from LN lesions, while other immune cells are present, but unable to eliminate cancer cells (not shown). Some cancer cells may exit through the efferent lymphatic vessel and seed secondary draining LNs. Recent evidence suggests LEC sphingosine-1-phosphate (S1P) helps shape the antitumor immune response.

**Figure 2:**
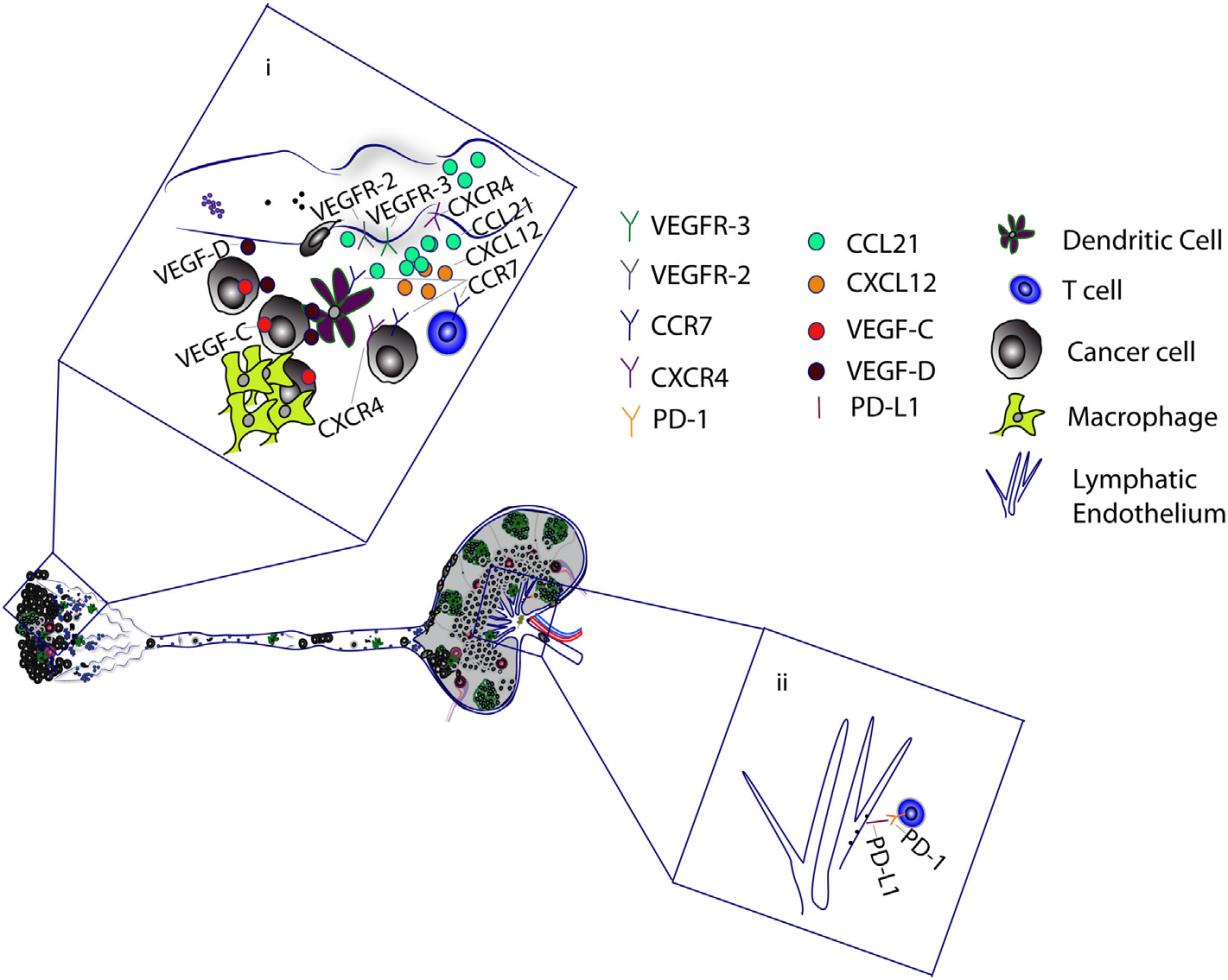
Tumor-associated lymphatic vessels promote metastasis and cancer progression. (i) Tumor-associated macrophages and cancer cells secrete VEGF-C and VEGF-D, which binds to VEGFR-2/3 on lymphatic capillaries to mediate lymphangiogenesis. VEGF-C upregulates CCL21 production by lymphatic endothelial cells (LECs). CCL21 attracts cancer cells, T cells, and dendritic cells (DCs), which express CCR7, a receptor for CCL21. VEGF-C has also been shown to upregulate CXCR4 expression on LECs. The CXCL12–CXCR4 axis can stimulate lymphangiogenesis to promote cancer cell migration. Alternatively, LECs promote the migration of CXCR4-positive cancer cells by secretion of CXCL12. Tumor antigen is delivered to the tumor-draining lymph nodes, where it is presented to T cells by DCs and LECs. (ii) Binding of LEC programmed death-ligand 1 (PD-L1) with T cell PD-1 receptor induces CD8 T cell tolerance to tumor antigens.

Metastasis is the leading cause of death from cancer (1) and represents a challenging clinical problem. Lymph nodes (LNs) are common sites of metastasis and nodal disease predicts increased mortality in many cancer types. Meanwhile, LNs are critical for initiating antitumor immune responses. Thus, cancer cells that have metastasized to LNs must escape immune detection to avoid destruction. The process of lymphatic metastasis is regulated at several steps and by several different molecules (Figures 1 and 2, respectively), beginning with the orchestration of lymphangiogenesis and preparation of a LN microenvironment favorable for tumor growth (premetastatic niche). Cancer cells then invade tumor-associated lymphatic vessels at the primary site *en route* to tumor-draining LNs (TDLNs), where they survive and grow. In a metastatic node, immunological destruction of cancer cells depends on the degree of cancer cell immunogenicity and the extent of nodal immunosuppression. Similar to primary tumors, cancer cells in LNs shape their interactions with the host immune system by controlling the infiltration and reactivity of immune cells. The local microenvironment of the LN also dictates the growth and response of LN metastases to therapeutic intervention. For example, only a small fraction of drugs delivered systemically accumulate in LNs (2). Identifying effective therapy for LN metastases takes on new urgency as cancer cells in LNs have also been proposed to disseminate to other metastatic sites by lymphatic or hematogenous routes. In this review, we summarize recent progress in the understanding of lymphatic metastasis and metastatic outgrowth. We also discuss the consequences of lymphatic metastasis and therapeutic efforts to target LN lesions in experimental mouse models and humans.

## Lymphatic Endothelial Cells (LECs) and Tumor Immunity

### Mediators of Immunosuppression

Recent studies suggest that in addition to serving as a portal for tumor dissemination, lymphatic vessels facilitate tumor growth through immune suppression (3). To generate an antitumor T cell response, migratory dendritic cells (DCs) from primary tumors cross-prime naïve T cells in TDLNs (4). The adhesion ligand Mac-1 on DCs can bind to the adhesion molecule intercellular adhesion molecule-1 (ICAM-1), which is upregulated on endothelial cells of collecting lymphatic vessels in response to inflammation (5), including inflammation generated by the tumor microenvironment. This Mac-1/ICAM-1 interaction inhibits DC maturation (5) and may blunt the ability of DCs in an inflamed tumor microenvironment to prime antitumor T cells. LECs further inhibit antitumor T cell responses by inducing tolerance to tumor antigens. LECs can scavenge tumor and other peripheral antigens and cross-present them to CD8 T cells, but LECs lack co-stimulatory molecules needed for full activation of CD8 T cells (6). Programmed death-ligand 1 (PD-L1), a ligand for the T cell inhibitory receptor PD-1, is expressed on tumor-associated lymphatic vessels (7) and LN LECs (8). The engagement of PD-L1 on LECs with T cell PD-1 induces CD8 T cell tolerance to tumor antigens (7, 8). Given the paucity of antitumor T cells and functional lymphatic vessels within some tumors (9, 10), the degree of CD8 T cell interaction with tumor-associated lymphatic vessels and their degree of inhibiting antitumor immunity is unclear. Recent studies show that the presence of LECs inside tumors make the tumors more responsive to anti-PD-1 therapy, suggesting lymphatics can have a potent inhibitory effect on T cell function (11).

In normal physiology, LECs produce sphingosine-1-phosphate (S1P), which is secreted into lymph and controls lymphocyte exit from LNs (12). Neutralization of systemic S1P with a therapeutic antibody suppresses lung metastasis (13). More recently, a genome-wide functional screen identified the S1P transporter spinster homolog 2 (*Spns2*) as a regulator of metastatic colonization in animals with experimental lung metastases (14). Spns2 is expressed on LECs and is critical for LEC release of S1P (15). Global and lymphatic-specific deletion of *Spns2* decreased pulmonary metastases following intravenous tumor cell injection, with fewer total T cells present in lungs relative to WT mice (14). However, a higher proportion of effector memory T cells to regulatory T cells (Tregs) were found in the lungs of *Spns2* deficient animals (14). Coupled with enhanced KLRG1^+^, CD69^+^CXCR3^+^ T cell activation, these findings were suggestive of an adaptive immune response against lung metastases (14). In addition, the natural killer (NK) cell population in the lungs of *Spns2* deficient animals was increased and limited the growth of lung metastases, even after CD8 T cell depletion (16). By contrast, S1P signaling has the potential to promote antitumor T cell responses. LEC- produced S1P appears to function not only as a regulator of lymphocyte circulation, but also supports naïve CD4 T cell survival by maintaining their mitochondrial content through an S1P_1_ receptor-dependent mechanism (16). Targeting S1P signaling to decrease metastatic burden requires a better understanding of its temporal and spatial role in shaping antitumor T cell responses.

The chemokine CCL21 is produced by LECs and mobilizes DCs to LNs (17). Although CCL21 can also establish an immunosuppressive tumor microenvironment (18), it was recently shown that tumor-associated lymphatic vessels also facilitate naïve T cell recruitment into melanoma tumors through CCL21 production (11). The presence of T cells allows local immune priming and the ability to unleash potent antitumor activity in response to vaccination or immune checkpoint blockade (11). So while LECs and CCL21 themselves help suppress antitumor immune responses, their ability to recruit T cells sensitizes lymphangiogenic tumors to immunotherapy. Cancer patients with elevated VEGF-C (indicative of increased lymphangiogenesis and lymphatic metastasis) typically have poor prognosis. Based on recent data, VEGF-C [which upregulates CCL21 (19)] may now be used as a biomarker in these patients to predict their response to immunotherapy. The ability of T cells with effector function to leave primary tumors and travel to TDLNs through afferent lymphatic vessels also suggests that tumor-associated lymphatic vessels may assist in dampening tumor growth (20).

The above examples illustrate the potential for context-dependent benefit of inhibiting, altering or utilizing intrinsic properties of LECs to maximize effective antitumor immune responses.

### Lymphangiogenesis

Lymphangiogenesis is a hallmark of many solid tumors. The expansion of the lymphatic network is primarily mediated by VEGF-C and its receptors VEGFR-2/3 (21). VEGF-D also binds VEGFR-2/3 and potently induces lymphangiogenesis (22, 23). Both VEGF-C and VEGF-D expression correlate with increased LN metastasis (24). Furthermore, *Vegf-d* deficient mice displayed less lymphatic metastasis relative to tumor-bearing wild type mice (25). Many preclinical studies have shown prevention of lymphatic metastasis by blocking VEGF-C or VEGF-D-mediated lymphangiogenesis (26–28). Clinically, this point of intervention is challenging as lymphangiogenesis is an early event in the natural history of cancer progression and many patients will already have LN metastases on initial presentation. However, additional opportunities may exist to target lymphangiogenesis. For example, lymphangiogenesis was identified as a mechanism of tumor resistance to paclitaxel chemotherapy in mice (29). In response to paclitaxel in mouse models, tumor-infiltrating macrophages secrete cathepsin, which activates heparanase. Active heparanase, by unknown mechanisms, increases both VEGF-C transcription and tumor invasiveness (30). In another study, VEGFR-3 reporter mice were used to image lymphangiogenesis in distant LNs, liver, lungs, and spleens in tumor-bearing mice. Following tumor resection, VEGFR-3 levels declined but reemerged before tumor relapse, suggesting a defined window of opportunity to inhibit lymphangiogenesis in distal premetastatic organs (31).

Although there are a plethora of targets that exist to inhibit lymphangiogenesis (32), many studies find that VEGF-C/-D-VEGFR-2/3 signaling directly or indirectly promotes lymphangiogenesis in response to a wide range of stimuli (22, 24, 25). Surprisingly, few clinical trials targeting the lymphatic endothelium in cancer are ongoing, although several small molecules that non-selectively target VEGFR-3-mediated lymphangiogenesis are approved for cancer indications (33). A Phase I study was recently completed that assessed the effect of LY3022856 (IMC-3C5), a monoclonal antibody targeting human VEGFR-3, on colorectal cancer (CRC) (34). While LY3022856 was well tolerated at the given dose, minimal antitumor benefit was noted in patients with CRC. The impact of LY3022856 on tumor lymphangiogenesis and lymphatic metastasis was not assessed. As mentioned earlier, inhibiting lymphangiogenesis through targeting VEGF-C/D or VEGFR-3 is also complicated by the uncertainty of the effect that lymphatic vessels have on antitumor T cell responses (11).

Independent of lymphatic vessel growth and lymphangiogenesis, VEGF-C can promote cancer metastasis by disruption of the vascular endothelial cadherin/β-catenin complex at intercellular junctions of LECs (35). The authors concluded that enhanced permeability of intestinal lymphatic vessels caused by VEGF-C can increase CRC transmigration and metastasis. Thus, how therapies targeting VEGF-C/D signaling will impact cancer progression will depend on the specific context of the disease as well as other therapies being used in conjunction.

## Establishment of Premetastatic Niche in TDLNs

### Extracellular Vesicles (EVs)

In addition to the delivery of cells, lymphatic vessels deliver primary tumor-derived soluble and vesicle-associated factors to condition TDLNs before the arrival of cancer cells. Exosomes, a type of EV, were shown to modulate the immune and stromal response in TDLNs (36, 37). Melanoma cells injected into the footpad and taken up by local lymphatic vessels had a similar distribution pattern as premetastatic melanoma-derived exosomes previously injected into the footpad, suggesting exosomes influence the recruitment of cancer cells to the LN (38). Mechanistically, exosomes upregulated host genes that promoted the retention, recruitment, and progression of LN metastases (38). It is unclear what components of exosomal cargo (e.g., mRNA, miRNA, or proteins) were necessary for changes in nodal gene expression. Melanoma exosomes have also been shown to enhance metastasis by “educating” and mobilizing bone marrow-derived cells to metastatic sites (36), including LNs, where they facilitated cancer cell invasion. Melanoma-derived EVs were identified in afferent lymphatic vessels of patients (39). Cocultures of EVs from human melanoma cells with DCs resulted in inhibition of DC maturation (39). In premetastatic LNs, CD169^+^ subcapsular sinus (SCS) macrophages capture tumor-derived extracellular vesicles (TeVs) (40) and protect host LNs from TeV-mediated immunosuppression. TeV release was required to accelerate tumor progression after the investigators depleted host macrophages. It is, however, unclear how TeVs can escape capture by SCS macrophages to deliver their payload under normal conditions. In contrast to the pro-tumor effects of EVs, migratory DCs acquire tumor-secreted vesicles released by circulating tumor cells in the lung (41). From here, the vesicle-loaded DCs migrate to mediastinal LNs to interact with and potentially activate antitumor T cells to limit metastatic growth. Taken together, these data suggest that TeVs have immune regulatory functions as well as help initiate and support the growth of LN metastases.

### Lymphatics in a Premetastatic LN

It is known that lymphangiogenesis occurs in premetastatic LNs (42, 43). Nodal lymphangiogenesis has been shown to be tumor antigen independent and B cell dependent (42, 44) through production of VEGF-A and VEGF-C (43–46). Recently, midkine, a heparin-binding factor produced by tumor cells, was identified as a critical factor for mTOR-dependent lymphangiogenesis in premetastatic sites including skin, LN, spleen, and lung (31). Furthermore, midkine mediated tumor cell adhesion to LECs and promoted tumor colonization in distant organs.

It is unclear how LN lymphangiogenesis results in metastasis. One hypothesis is that LN lymphangiogenesis may lead to more efficient delivery of cancer cells to LNs and distant organs (47). This may be facilitated by the increased lymph flow that accompanies increased LN lymphangiogenesis (42). Increased lymphatic drainage from primary tumors was also associated with LN enlargement (48) and nodal remodeling, which may alter the distribution of antigen and soluble factors. Increased lymphatic drainage also coincided with collagen and hyaluronic acid deposition in premetastatic TDLNs of B16F10 tumors. Parental B16 melanoma cells failed to increase TDLN matrix remodeling and were inefficient at metastasis, suggesting that in addition to lymphangiogenesis, an increase in TDLN matrix may be a prerequisite for formation of LN metastases (48, 49).

### Fibroblast Reticular Cells

The LN contains an array of stromal cells, including fibroblastic reticular cells (FRCs). Much is known about the tumor-promoting effects of cancer-associated fibroblasts, but few reports have characterized the FRC response to cancer cells. Transcriptional profiling of FRCs from non-tumor-bearing animals revealed abundant expression of chemokines critical for lymphocyte recruitment, including *CCL19, CCL21, CXCL12*, and *CXCL13* (50). FRCs also produce several forms of collagen (50), indicative of their role in forming the conduit system that delivers small antigens deep into the LN for antigen presentation (51). FRCs express genes necessary for MHC class 1/2 presentation (50) and can present peripheral antigens to T cells (52). Similar to LECs, FRCs contribute to peripheral tolerance by facilitating deletional tolerance (52, 53) and dampening effector T cell proliferation (54, 55). A recent transcriptional analysis revealed FRCs in premetastatic LNs are “reprogrammed” to favor tumor growth (56). In spontaneous and orthotopic models of melanoma, TDLN FRCs proliferated, but produced less *IL-7* and *CCL21*, which are critical for T cell survival and guidance, respectively. The reduction in *IL-7* and *CCL21* resulted in disruption of the TDLN architecture, with loss of clear delineation between B and T cell zones. In a separate study, the loss of FRC *CCL21* in the TDLN was associated with disorganized T cell and B cell zones in premetastatic LNs (57). The perturbation of LN architecture due to altered FRC signaling molecules suggests altered immune responses to tumors. Since LNs are priming sites for adaptive immune responses, the disordered LN architecture may fail to elicit systemic protection from subsequent heterogeneous cancer cell clones that arrive in the TDLN (56). In metastatic LNs, collagen production was increased relative to tumor-free LNs (58). Although unclear whether recruited fibroblasts, FRCs, or cancer cells are the source of additional collagen, the investigators speculate that the increased density of collagen fibers may allow cancer cells to adhere and migrate within metastatic LNs. It is unknown how tumor cells influence FRC transcriptional status.

## Tumor Cell Migration to LNs

Cancer cells enter lymphatic vessels and travel with the lymph to establish LN metastasis (59). Cancer cells may actively migrate into lymphatic capillaries in response to molecular cues (19, 60) or they may passively enter into lymphatic capillaries (19, 60). Metastasis to the LN likely depends on a combination of intrinsic cancer cell properties and signals in the tumor microenvironment. VEGF-C and lymphatic flow both upregulate CCL21 in lymphatic endothelium (19, 61), attracting CCR7^+^ tumor cells (62). In a triple-negative breast cancer model, CCL21 was sufficient to recruit RORγt^+^ innate lymphoid cells (ILCs) into the primary tumor and promote metastasis to LNs (63). Furthermore, CXCL13 was required for clustering of ILCs and induction of epithelial–mesenchymal transition, likely driving invasion of cancer cells. In breast cancer patients, the presence of ILCs was significantly associated with lymphatic invasion at the primary tumor.

Several studies have shown that another chemokine, CXCL12, facilitates lymphatic metastasis of CXCR4^+^ tumor cells (64–66). CXCL12 expression is found on lymphatic vessels within primary tumors and guides CXCR4*^+^* melanoma cells toward lymphatic vessels. Migration and invasion of CXCR4^+^ papillary thyroid carcinoma cells are dependent on CXCL12, which was produced by senescent cancer cells at the invasive border (67). These senescent cells invaded lymphatic vessels and persisted in metastatic foci, suggesting that they may promote lymphatic metastases. CXCR4 is also expressed on the surface of LECs (68) and is critical for lymphangiogenesis through CXCL12 stimulation, independent of the VEGFR-3 pathway (68). Thus, targeting the CXCR4/CXCL12 may provide a dual benefit of inhibiting cancer cell migration and lymphangiogenesis to curb lymphatic metastasis.

After entry of cancer cells into lymphatic vessels, it is thought that lymph flow allows cancer cells to traverse the collecting lymphatic vessel network until they reach TDLNs (59). Based on 3D modeling, it was predicted that smaller breast cancer cells may have a survival advantage over larger breast cancer cells in the lymphatic circulation because of the lower wall shear stress that they encounter (69). Several studies have shown that inflammation causes dilation and inhibits contractile ability of collecting lymphatic vessels (70, 71). More work needs to be done to determine if tumor-induced collecting lymphatic dilation (10, 22, 59) or reduced contraction (72) enhances tumor cell dissemination by decreasing the shear stress on cancer cells. It is known that tumor cells can arrest within lymphatic vessels while “in-transit” to LNs (73). Compromised barrier integrity of lymphatic vessels may allow arrested cancer cells to escape lymphatic vessels and form metastases (74, 75). Additional characterization of the mechanism of how tumor cells attach to lymphatic endothelium and grow within lymphatic vessels is needed to treat in-transit metastases.

Recently, the chemokine CCL1 and its receptor CCR8 were demonstrated to be important for melanoma cell entry into TDLNs. CCL1 is produced by SCS LECs and mediated entry of CCR8^+^ melanoma cells into LNs (60). Tumor cells in the SCS can also bypass the LN parenchyma and drain through cortical and medullary sinuses to exit LNs *via* efferent lymphatic vessels (76). The enzyme lipoxygenase 15 (ALOX15) metabolizes arachidonic acid to 12(S)-hydroxyeicosatetraenoic acid [12(S)-HETE] and 15(S)-hydroxyeicosatetraenoic acid [15(S)-HETE]. Cancer cell-derived 12(S)-HETE forms discontinuities in the walls of lymphatic vessels, allowing LN metastases to invade nodal lymphatic vessels (77). The fate of these cancer cells is unclear, although TDLN lymphangiogenesis has been reported to be involved in further lymphatic spread of human breast cancer (78) and the presence of lymphatic vessel invasion by LN metastases is associated with worse survival (79). It is possible that cancer cells circulate to additional nodes through lymphatic vessels and eventually enter the systemic circulation through the thoracic duct.

## Immune Evasion in TDLNs

### Macrophages

Lymph node SCS macrophages are the first line of defense against tumor cells entering the LN. SCS macrophages capture microbes, antigen–antibody complexes and dead cancer cells for delivery of these antigens to nearby immune cells (80, 81). In premetastatic LNs, an experimental antigen (a fluorescent protein overexpressed in tumor cells) from the primary tumor was captured by SCS macrophages and distributed to follicular DCs, resulting in antibody production against the antigen (82). SCS macrophages can also directly cross-present tumor antigens to CD8 T cells (81). Sinus macrophages in regional LNs of CRC patients made direct contact with CD8 T cells and a high density of sinus macrophages is associated with increased overall survival (83). On the other hand, tumor-associated macrophages are often associated with poor prognosis and promotion of tumor growth (84). Strategies to deplete TAMs include targeting colony-stimulating factor 1-receptor (CSF1-R) (85), which controls macrophage chemotaxis. Interestingly, an increase in the burden of LN metastases was found following treatment with an anti-CSF1-R antibody (86). This increase in metastatic burden was associated with the loss of SCS macrophages due to anti-CSF1-R therapy (86). Tumor-promoting (M2) macrophage depletion strategies should examine the effect on SCS macrophages to avoid unintended growth of LN metastases.

### Neutrophils

Neutrophils, such as macrophages, are heterogeneous and have been reported to have either pro-tumor or antitumor phenotypes in primary tumors (87). High levels of tumor-associated neutrophils are associated with LN metastasis and poor prognosis (88). Granulocyte colony-stimulating factor was necessary to expand and polarize neutrophils to an immunosuppressive phenotype in mice bearing mammary tumors (89). The immunosuppressive neutrophils, whose expansion was also driven by IL-17, were able to suppress cytotoxic T cells and facilitate the establishment of LN metastases. Neutrophils can also secrete pro-inflammatory leukotrienes and initiate LN metastases *via* leukotriene receptors (LTR) on cancer cells (90). LTR expression was found in a cohort of primary and LN tumors from breast cancer patients. More research is needed to characterize the phenotype of neutrophils found in LN metastases and their role in metastatic progression.

### T Cells

The TDLN often fails to produce effective antitumor immunity and instead tolerizes the patient to tumor antigens (91). The mechanisms that induce systemic tolerance include cross-presentation of tumor antigen by tolerogenic antigen-presenting cells, apoptosis of antigen-presenting cells (92), and suppression of antitumor T cells by an expanded pool of Tregs. Experimentally, subcutaneous injection of B16 melanoma resulted in tolerized CD8 T cells and lethal metastatic outgrowth (93). However, B16 cells implanted directly into LNs—without a primary tumor—were rejected, supporting previous evidence (94) that showed the primary tumor exerts a tolerogenic effect on the TDLN (94). However, initial metastatic deposits in lymphoid organs are important for the induction of antitumor CD8 T cells and tumor rejection (94). Notably, tumor cells injected into LNs using different cancer models have been shown to persist and disseminate to distant organs (95). These differences may be explained by factors such as the immunogenicity and antigen presentation capabilities of different cancer cells. Increased LN metastasis was found in cancer patients with tumor downregulation of MHC I (96).

### NK Cells

Natural killer cells are cytotoxic lymphocytes of the innate immune system that are often recruited to tumors including prostate (97), melanoma, kidney, liver, and breast (98). NK cells are able to recognize and eliminate cells with aberrant ligand or altered/absent MHC expression (99, 100). However, NK cell infiltration into primary tumors is limited; NK cells that enter tumors are often found in primary tumor stroma and lack direct contact with cancer cells (98). Likewise, NK cells in metastatic LNs were adjacent to metastatic melanoma lesions (101). Moreover, NK cells isolated from metastatic human LNs showed significantly reduced cytotoxicity (101, 102). NK cells that were isolated from metastatic LN lesions and stimulated with IL-2 or IL-15 displayed more efficient lysis of cancer cells (101), suggesting that the LN tumor microenvironment suppresses NK cell function. Thus, despite their presence in the TDLN, immunosuppressed NK cells may lack the ability to eliminate cancer cells.

### B Cells

The number of B cells in premetastatic TDLNs is significantly increased (42). EVs from tumor cells increased immunosuppressive B cells in premetastatic LNs (40). Depletion of SCS macrophages in tumor-bearing animals permitted interactions of TeVs with B cells and led to increased antibody production. Although the tumor antigen specificity of the antibodies are unknown, transfer of these antibodies to wild-type recipient mice correlated with enhanced tumor growth compared with antibody transfer from tumor-bearing animals without disruption of SCS macrophages. Regulatory B cells (Bregs) were recently identified in mouse and human blood and secondary lymphoid organs (103). Bregs can secrete immunosuppressive cytokines, such as TGF-β and IL-10 (104), to dampen the effector activity of antitumor T cells. However, the data conflict on whether Bregs support or suppress tumor growth (105). The phenotypic markers that identify Bregs also remain unclear.

Together, these data suggest that multiple immune cell types in premetastatic and metastatic LNs have suboptimal killing activity for cancer cells. Identifying molecular targets to reverse immune suppression of several cell types will be of therapeutic benefit in treating metastatic cancer.

## Ectopic ln Immunity

High endothelial venules (HEVs) and their homeostatically associated chemokines are crucial for entry of naïve lymphocytes into LNs (106). Ectopic HEVs in primary human breast cancer and melanoma tumors allow lymphocytic intravasation and predict a favorable prognosis (106, 107). LTα_3_–TNFR signaling, not LTβR, was critical for the generation of HEV-like vasculature expressing peripheral node addressin (PNAd) in models of lung cancer and melanoma (108) studied by Peske et al. The PNAd^+^ vasculature was critical for infiltration of naïve T cell into tumors. Growth of HEV-containing tumors was delayed although treatment with the S1P antagonist FTY720 retained tumor-specific T cells in secondary lymphoid organs (108). These data suggest that naïve T cells can differentiate into effector T cells within primary tumors.

Tertiary lymphoid organs (TLOs) are aggregates of immune cells that mimic the structure and function of LNs. They are formed in several diseases associated with chronic inflammation, including cancer (109). TLOs include lymphocytes, lymphatic vessels, and HEVs (110). TLOs within tumors are associated with improved outcomes for patients and function as sites of immune priming for the generation of antitumor lymphocytes. In animal models of pancreatic and breast cancer, tumor-associated blood vessels developed HEV markers and formed TLO-like structures in response to a combination of antiangiogenic (DC101) and immune checkpoint (PD-L1) therapy (111). Formation of LTβR signaling-dependent HEVs resulted in enhanced infiltration and activation of antitumor T cells, and better antitumor responses (111). By contrast, the presence of TLOs and ectopic HEVs has been shown to promote tumor growth in other experimental conditions. Finkin et al. found that the cytokine-rich environment of liver TLOs promoted the growth of hepatocellular carcinoma progenitor cells in a hepatocellular carcinoma model (112). From studies using a murine model of lung cancer, Joshi et al., found that TLOs in tumor-bearing lungs were a site of antigen presentation (113). However, the abundant Tregs within the TLOs presumably suppressed antitumor T cell responses. The use of immune checkpoint inhibitors, such as the anti-CTLA-4 antibody ipilimumab, can lead to the depletion of Tregs (114) and may enhance the antitumor function of effector lymphocytes in tumors and TLOs with a high Treg: effector T cell ratio. In a separate study, depletion of Tregs resulted in intratumoral HEV formation and higher numbers of activated CD4 and CD8 T cells in carcinogen-induced fibrosarcomas, resulting in reduced tumor burden (115, 116). TNF-producing T cells that signaled through TNFR were critical for intratumoral HEV neogenesis (116).

Given the context-dependent benefit of TLOs, it is unclear whether induction of TLOs is a viable therapeutic strategy to limit tumor progression. TLO formation might need to be accompanied by a strategy to prevent Treg formation to generate an effective antitumor response. Moreover, the signaling mechanisms to induce TLO formation appear to be dependent on various cytokines and receptors of the lymphotoxin/TNF family as well as the local microenvironment, requiring further research to build our understanding.

## Growth of Cancer Cells in LNs

### LN Vasculature

In primary tumors, hypoxia is associated with expression of VEGF, which in turn leads to the sprouting of nascent blood vessels. Primary tumor hypoxia has been shown to increase the frequency of LN metastasis (117) by upregulating the integrin α_5_ subunit, which is required for 3D cell migration *in vitro* (117). We, and others, have found hypoxic tumor cells in the LN (118, 119). It is unclear whether these hypoxic cancer cells in the LN sinus maintain this status from their state in the primary tumor or if tumor cells become hypoxic on arrival and proliferation in the avascular LN sinus.

Angiogenic and non-angiogenic-dependent metastases have been found in LNs and other metastatic tissues, such as the lung and liver (120–123). The presence of hypoxic cancer cells correlates with endothelial cell proliferation in some LN metastases, a pattern that could be predicted by the characteristics of the primary tumor (124). We found elevated VEGF and angiogenesis in primary tumors but did not find the same in metastatic LNs, despite the presence of hypoxic cancer cells (118). In agreement with the findings of the lack of angiogenesis in LN metastasis from our laboratory, other studies have shown that the vascular density of metastatic LNs is lower than that of non-metastatic nodes (121, 125, 126).

Although overexpression of VEGF leads to the expansion of the LN lymphatic vessel network (43–45, 127), a limited number of studies suggest VEGF, or other growth factors, have an effect on the number of blood vessels within the LN. Overexpression of VEGF has been reported to increase the number of HEVs (45). By contrast, other reports demonstrate that VEGF only increases the diameter of LN blood vessels, perhaps through proliferation of endothelial cells of existing vessels (43, 127). The scarcity of evidence concerning VEGF and inflammation-induced sprouting angiogenesis relative to lymphangiogenesis raises questions about the mechanistic control of the LN vasculature. During the progression of LN metastases, the existing LN vasculature may be sufficient to support tumor growth. It has been proposed that angiogenesis is redundant for tumor growth in the LN due to the rich native vascularity of LNs; the vessel density of the LN is comparable to that of the primary tumor (125). It has also been proposed that remodeled HEVs in TDLNs can nurture established metastatic lesions in LNs (128). Although studies of VEGF in other metastatic organs require investigation, the unresponsiveness of LN metastases to antiangiogenic therapy (59, 118) adds another explanation for the poor outcomes of anti-VEGF therapy in adjuvant settings.

Recent studies have investigated mechanisms of resistance to antiangiogenesis therapy in metastatic disease. The tyrosine kinase inhibitor sunitinib, whose targets include VEGF receptors, stimulated transcription and mRNA stabilization of *VEGF-C* in a xenograft model of renal cell carcinoma (129). As a result, lymphangiogenesis and lymphatic metastasis were increased. A recent study identified vascular cooption in breast cancer liver metastases as another resistance mechanism to anti-VEGF therapy (123). In a “replacement” pattern of growth, liver metastases replaced hepatocytes and were physically associated with liver sinusoidal blood vessels. Silencing of Actin Related Proteins 2/3 (ARP 2/3), which mediate breast cancer cell motility, effectively inhibited vascular cooption of these liver metastases. It remains to be determined if this mechanism of vascular cooption is active in LN metastases. As multiple modes of growth were seen in liver metastases, metastatic growth and vascularization may depend on multiple factors, including the tumor type. Further investigation is needed to tailor treatments targeting the growth of metastases, including LN metastases.

## Clinical Management and Treatment of LN Metastasis

Lymph node metastasis is a critical prognostic indicator for patients with solid tumors including melanoma, breast, and gastric cancers. However, the role LN metastasis plays in cancer progression has been debated in the clinic for decades (130). The guidelines and treatment strategies for patients with nodal disease are evolving as clinical trials are conducted to improve the ability to contain and treat metastases. Until recently, axillary LN dissection (ALND) had been the standard of care for breast cancer patients with sentinel-node involvement. However, recent clinical trials designed to define the benefit of ALND has changed the way breast cancer patients are being treated. The International Breast Cancer Study Group Trial 23-01 (IBCSG 23-01) clinical trial was conducted to determine the benefit of ALND in patients with limited sentinel-node involvement (1–2 micrometastatic nodes) and tumors less than 5 cm (131). Five-year follow-up showed no disease-free survival benefit in patients that underwent ALND compared with those that did not. These findings are consistent with the ACOSOG Z0011 trial involving patients with limited sentinel-node involvement undergoing breast-conserving surgery, in which these patients were assigned randomly to receive either ALND or no further axillary surgery (132, 133). Both trials suggest that ALND in early-stage breast cancer patients with limited nodal involvement do not have a survival benefit compared with patients that do not undergo ALND. These trials have led to a reduction in the number of breast cancer patients undergoing ALND, which has also reduced the morbidities associated with ALND. However, all of these patients received traditional systemic adjuvant therapy and radiation that potentially eliminated any residual disease in the LNs (132). Thus, radiation and systemic therapies may be sufficient to control nodal disease, making ALND unnecessary for breast cancer patients with limited LN involvement.

To test this hypothesis, recent clinical trials have assessed the benefit of axillary radiation therapy (ART) in early-stage breast cancer patients as an alternate treatment strategy to ALND. Although ALND provides excellent regional control of the disease, patients experience debilitating side effects such as lymphedema. The After Mapping of the Axilla: Radiotherapy or Surgery (AMAROS) randomized phase III trial compared sentinel-node-positive T1-2 breast cancer patients who received either ART or ALND as adjuvant treatment after systemic therapy (134). The results from this trial showed that ART provided excellent local control of disease and the outcomes were comparable to ALND. Furthermore, ART patients had fewer complications compared with those receiving ALND.

For melanoma patients, the Multicenter Selective Lymphadenectomy Trial-I (MSLT-I) provided evidence that patients who undergo sentinel LN (SLN) biopsy have an increased survival rate (135). In this trial, patients were randomized to either (i) wide excision of the melanoma with SLN procedure, followed by complete nodal dissection in patients with a positive SLN or (ii) wide excision and nodal observation, with lymphadenectomy at the time of LN recurrence. However, the importance of complete LN dissection remained controversial, as the main difference between the treatments for patients with disease in LNs was the timing of when lymphadenectomy occurred, with patients having disease removed earlier (SLN patients) having better outcomes. More recently, results of the MSLT-II, a randomized, multicenter, phase-3 clinical trial conducted on 1,934 melanoma patients were reported (136). The MSLT-II trial evaluated the importance of complete LN dissection by randomizing patients with sentinel-node metastases to either immediate complete LN dissection or nodal observation with ultrasonography. Results from this trial showed that immediate complete LN dissection in patients with sentinel-node metastases was not associated with increased 3-year melanoma-specific survival. However, patients that underwent complete LN dissection had increased rates of local-disease control compared with the observation group at 3 years (92 + 1.0 vs. 77 + 1.5%) and a slightly higher rate of disease-free survival compared with the observation group at 3 years (68 + 1.7 and 63 + 1.7%, respectively). The results of the MSLT-I and MSLT-II trials show that removing positive SLNs improves outcomes, but that further LN removal does not improve 3-year overall survival. However, complete nodal dissection in positive SLN patients did improve disease recurrence. The importance of recurrent disease in cancer progression and ultimate patient survival is thus being called into question. It will be critical to address whether there is a difference in 5- and 10-year overall survival in the MSLT-II trial, as these data may be able to account for systemic progression from the recurrent disease. The risk of systemic progression from recurrent disease must be weighed against the very clear reduction in the rate of lymphedema in patients that did not undergo complete LN dissections.

Taken together, the results from these clinical trials have revolutionized the way clinicians manage cancer treatment. Current clinical practice has adopted the theory that less extensive LN dissections for patients with early-stage disease reduce complications without changing overall survival. In these cases, systemic adjuvant therapy and radiotherapy are able to manage any residual disease. Treating local disease in the LN that has not yet progressed to other locations with systemic therapies and radiation could decrease the long-term mortality rate in patients (137), potentially mitigating the impact of less extensive surgeries that leave some LNs with metastatic cancer cells in patients. However, fundamental questions in the biology of LN metastases still remain unanswered, including: What is the fate of cancer cells once they metastasize to the LN? Do LN metastases spread beyond the node and contribute to disease progression? Answers to these questions through continued research will give us better insights into the most effective strategy to manage the progression of solid tumors.

Although there is no direct experimental evidence to show that cancer cells can escape the LN, a few genetic studies provide evidence that nodal disease can colonize distant organs. A recent study used phylogenetic tracing to analyze tumor cell evolution in CRC patients. The analyses from this study revealed that 35% of distant liver metastasis has LN metastasis as the closest phylogenetic neighbor (138). An earlier study characterized the somatic evolution of mutations in cancer cells from primary and metastatic tumors by genome sequencing in a genetically modified mouse model of small cell lung carcinoma (SCLC) (139). This analysis suggested that multiple related primary tumor subclones can seed the LN and a single clone can then spread further from the node to the liver. From the experiments in the SCLC model, the authors were unable to conclude if LN metastases preceded systemic dissemination and if the LN microenvironment altered the genetic and epigenetic makeup of cancer cells before distant dissemination.

Even with radiation and systemic therapies, some patients still have LN recurrences. Systemic therapies that minimize toxicity while targeting disease in LNs represent a promising approach. Only a small fraction of systemically delivered chemotherapy drug accumulates in LNs (2). We have found that increased vascular permeability of LN blood vessels did not increase chemotherapy penetration into the LN parenchyma (140). Many current strategies focus on optimizing lymphatic delivery and retention properties of therapeutics that target tumor-associated lymphatic vessels (141). Targeted delivery of immunomodulatory agents or cancer cell-specific cytotoxic drugs into TDLNs can improve cancer vaccination strategies (142) and eradicate disease from LNs, respectively (141).

## Conclusion

A growing interest and ability to study LEC biology has provided enormous insights into the role lymphatic vessels play in tumor metastasis and cancer progression. While it is known that tumor-associated lymphatic vessels are a route of metastasis, it is now appreciated that tumor cells also use these vessels to establish immunological tolerance in TDLNs. Although antitumor immune responses can be generated locally in primary tumors, TDLNs serve as critical sites for antitumor immune responses. It remains unknown how the systemic adaptive immune response to cancer is shaped by immunosuppressed LNs. Several additional important areas of exploration remain, including understanding the influence of the LN microenvironment on cancer cell behavior and determining the contribution of LN metastases to distant organ metastasis, which at present remains controversial. Therapies targeting LN metastases must also consider enhancing antigen presentation to tumor-specific T cells. Moreover, therapies to activate tumor-specific T cells should be considered in parallel with strategies to break tolerance and other immunosuppression mechanisms. With continued research focus on the LN, we will gain deeper insights into mechanisms of immune evasion by cancer cells. A more thorough understanding of the molecular signals that facilitate tumor metastasis to TDLNs and beyond may also provide therapeutic targets to control the further dissemination of lymphatic metastases. With these continued advances, patient survival from metastatic cancer will continue to improve.

## Author Contributions

DJ, EP, and TP contributed to the writing of the manuscript.

## Conflict of Interest Statement

The authors declare that the research was conducted in the absence of any commercial or financial relationships that could be construed as a potential conflict of interest.
